# Mediation effect of maternal triglyceride and fasting glucose level on the relationship between maternal overweight/ obesity and fetal growth: a prospective cohort study

**DOI:** 10.1186/s12884-023-05716-0

**Published:** 2023-06-16

**Authors:** Yanmei Wan, Yixuan Chen, Xiaoxia Wu, Aiqi Yin, Fuying Tian, Huafan Zhang, Xuna Huang, Linlin Wu, Jianmin Niu

**Affiliations:** 1 Department of Obstetrics, Shenzhen Maternity & Child Healthcare Hospital, The First School of Clinical Medicine, Southern Medical University, Shenzhen, Guangdong China; 2 Department of Obstetrics and Gynecology, The Eighth Affiliated Hospital, Sun Yat-sen University, Shenzhen, Guangdong China

**Keywords:** Obesity, Large for gestational age, Triglyceride, Fasting plasma glucose, Mediation analysis

## Abstract

**Background:**

Previous studies have suggested that maternal overweight/obesity is asscociated with macrosomia. The present study aimed to investigate the mediation effects of fasting plasma glucose (FPG) and maternal triglyceride (mTG) in the relationship between maternal overweight/obesity and large for gestational age (LGA) among non-diabetes pregnant women.

**Methods:**

This prospective cohort study was conducted in Shenzhen from 2017 to 2021. A total of 19,104 singleton term non-diabetic pregnancies were enrolled form a birth cohort study. FPG and mTG were measured at 24–28 weeks. We analyzed the association of maternal prepregancy overweight/obesity with LGA and mediation effects of FPG and mTG. Multivariable logistic regression analysis and serial multiple mediation analysis were performed. The odds ratio (OR) and 95% confidence intervals (CIs) were calculated.

**Results:**

Mothers who were overweight or obese had higher odds of giving birth to LGA after adjusting potential confounders (OR:1.88, 95%CI: 1.60–2.21; OR:2.72, 95%CI: 1.93–3.84, respectively). The serial multiple mediation analysis found prepregnancy overweight can not only have a direct positive effect on LGA (effect = 0.043, 95% CI: 0.028–0.058), but also have an indirect effect on the LGA through two paths: the independent mediating role of FPG (effect = 0.004, 95% CI: 0.002–0.005); the independent mediating role of mTG (effect = 0.003,95% CI: 0.002–0.005). The chain mediating role of FPG and mTG has no indirect effect. The estimated proportions mediated by FPG and mTG were 7.8% and 5.9%. Besides, the prepregnancy obesity also has a direct effect on LGA (effect = 0.076; 95%CI: 0.037–0.118) and an indirect effect on LGA through three paths: the independent mediating role of FPG (effect = 0.006; 95%CI: 0.004–0.009); the independent mediating role of mTG (effect = 0.006; 95%CI: 0.003–0.008), and the chain mediating role of FPG and mTG (effect = 0.001; 95%CI: 0.000-0.001). The estimated proportions were 6.7%, 6.7%, and 1.1%, respectively.

**Conclusion:**

This study found that in nondiabetic women, maternal overweight/obesity was associated with the occurence of LGA, and this positive association was partly mediated by FPG and mTG, suggesting that FPG and mTG in overweight/obese nondiabetic mothers deserve the attention of clinicians.

## Background

Large for gestational age (LGA), defined as birthweight ≥ 90th percentile for gestational age, can cause several maternal and fetal complications [1]. LGA is associated with an increased risk of cesarean birth, protracted labor, uterine rupture, postpartum hemorrhage and infection in mothers, as well as with shoulder dystocia and brachial plexus nerve injury in newborns [2–4]. Macrosomic newborns are more likely than normal-weight newborns to be overweight and obese later in life [5], and LGA offspring have a higher risk of developing conditions related to metabolic syndrome, such as hypertension, glucose intolerance, and dyslipidemia [6].

Pregestational BMI was shown to have a strong influence on the lipid profile, insulin resistance, and fatty acids [7]. Obesity in pregnancy is associated not only with marked hyperinsulinemia (in advance of glucose dysregulation) and abnormal lipids but also with impaired endothelial function and inflammatory upregulation [8]. Maternal obesity, independent of gestational diabetes mellitus (GDM), is associated with excessive fetal growth [9, 10].

Gestational diabetes mellitus is considered the most important risk factor for macrosomia, with approximately 15–45% of macrosomic babies born to mothers with GDM [11]. Hyperglycemia is a recognized metabolic factor that can lead to an unfavorable intrauterine environment and ultimately to fetal overgrowth [12, 13]. Women with higher fasting plasma glucose (FPG) levels in the second trimester were reported to have increased risks of delivering LGA babies [14]. However, high plasma glucose levels in patients who do not meet the diagnostic criteria for GDM are also associated with macrosomia and excess fetal weight [12, 15]. From clinical practice experience, even good glycemic control does not necessarily prevent fetal growth acceleration, especially in obese women [16]. It has therefore been assumed that other nonglucose metabolic parameters, such as maternal hypertriglyceridemia, may also regulate the birth weight of newborns [17].

As a typical metabolic feature, it is necessary to gradually increase the lipid profile used for fetal growth and development [18]. In overweight and obese mothers with GDM, maternal triglycerides (mTG) are partially responsible for LGA infants despite good maternal glucose control during pregnancy [19]. It is also well documented that elevated maternal triglyceride levels are associated with fetal birth weight, leading to excessive fetal growth and ultimately macrosomia [20], even in nondiabetic mothers [21].

Higher fasting glucose levels and hyperlipidemia are well-known results of maternal overweight/obesity, as well as contributing factors for increased fetal size, implying that FPG and mTG might have mediating effects in these causal pathways. Based on the available evidence and current obesity epidemic, the hypothesis that the association of maternal BMI with LGA is mediated through maternal circulating fuels (FPG and mTG) during pregnancy is plausible and that further determining which circulating fuels are important mediators of these relationships is crucial. Considering the complex effect of GDM on lipid changes during pregnancy [22], this large, hospital-based cohort study focused on nondiabetic women. These findings might help to explain the mechanism by which maternal obesity leads to LGA in nondiabetic pregnancies.

## Method and materials

### Study population

**Fig. 1:**
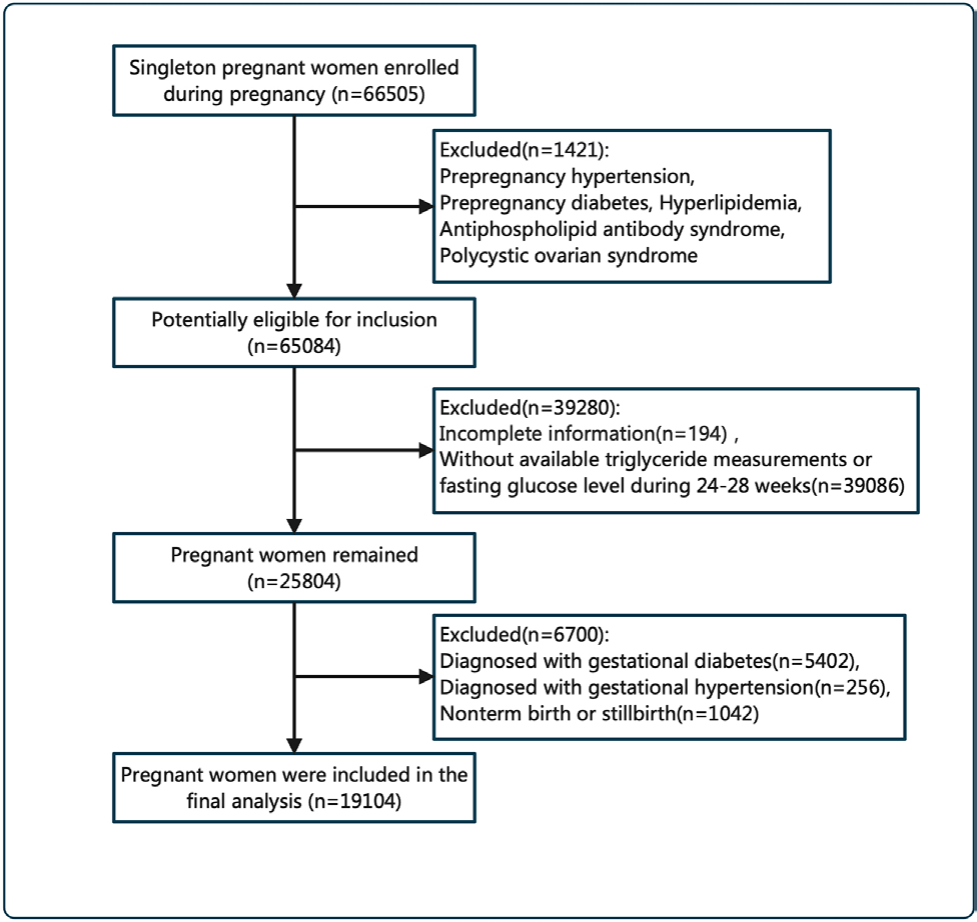
Flow chart of conclusion of study participants

In our study, all participants were recruited from Shenzhen Maternal and Childcare Hospital in Shenzhen, southern China. Pregnant women who had a singleton gestation were invited to join this study at their first prenatal clinic visit from 2017 to 2021 and intended to continuously receive prenatal care throughout pregnancy at the study hospital. Owing to missing data and some participants meeting the exclusion criteria, 19,104 pregnant women without diabetes who delivered term singleton babies and had complete information available were finally included in the analysis, and the reasons for exclusion were as follows: (i) diseases including prepregnancy hypertension and prepregnancy diabetes, hyperlipidemia, antiphospholipid antibody syndrome or polycystic ovarian syndrome (n = 1421); (ii) incomplete basic information (n = 194); (iii) no available triglyceride measurements or fasting glucose levels from 24 to 28 weeks (n = 39,086); (iv) meeting the IADPSG criteria [23] for GDM (n = 5402); (v) a diagnosis of gestational hypertension (n = 256); and (vi) nonterm birth or stillbirth (n = 1042). The study design and research flowchart are shown in Fig. 1. The study was conducted in accordance with the principles of the Declaration of Helsinki and granted by the Ethical Review Boards of Shenzhen Maternal and Childcare Hospital (Approval number: Shenzhen Maternal and Child Ethics Review No. 23).

### Data collection

Before data collection, written informed consent was obtained from all participants. Maternal demographic characteristics were collected through structured questionnaires at the first antenatal care visit. Research assistants collected data regarding education (schooling years ≤ 12 years or > 12 years), maternal age (< 35 years, ≥ 35 years), parity (primipara or multipara), alcohol consumption (yes or no), smoking (yes or no), Han nationality (yes or no), family history of diabetes (yes or no), and previous GDM history (yes or no). Maternal BMI was calculated based on measured weight (kg) and height (m) with light clothing and no shoes on at the first antenatal visit (before 12 weeks of pregnancy). Prepregnancy BMI was calculated based on height and prepregnancy weight (BMI = weight/height^2^), the exposure of interest in this study. Pregnant women were divided into four groups according to the prepregnancy BMI levels based on the Chinese criteria, with normal weight as the reference [24]: the underweight group (BMI < 18.5), normal weight group (18.5 ≤ BMI < 24), overweight group (24 ≤ BMI < 28) and obesity group (BMI ≥ 28). Maternal weight (kg) before delivery was obtained from electronic medical records. We subtracted the antenatal prepregnancy weight from the body weight at delivery as gestational weight gain. Clinical characteristics, including maternal medical disorders (e.g., the diagnosis of GDM) and laboratory tests, including fasting glucose levels and triglyceride levels from 24 to 28 weeks, were extracted from the electronic medical record system.

Infant characteristics, including sex, birth weight, gestational age at delivery, delivery mode (including vaginal delivery and cesarean section) and perinatal outcomes, were abstracted from neonatal medical records. Gestational age was calculated based on the date of the last menstrual period. When the difference between the gestational age calculated at the last menstrual period and the gestational age adjusted by early ultrasound examination was more than two weeks, the ultrasound results were used. According to birth weight and gestational age, newborns were classified into non-LGA (AGA and SGA) and LGA groups. Those with birthweights above the 90th percentile were classified as LGA, and their birth weights below the 10th percentile were defined as SGA, based on gestational age birthweights from NICHD [25]. AGA was defined as birth weights that met or exceeded the 10th percentile and fell below the 90th percentile for gestational age.

### Statistical analysis

**Fig. 2:**
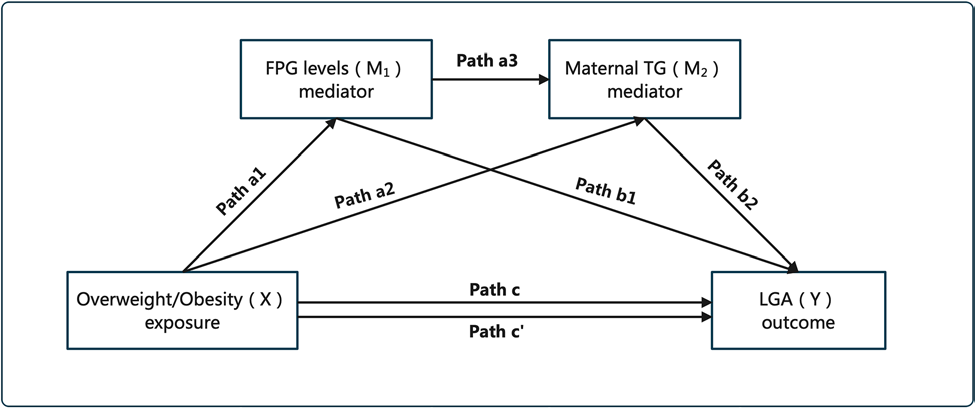
Serial meditation model for maternal overweight/obesity, FPG levels, mTG and LGA

The data for baseline characteristics of the participants are presented as the means ± SDs for continuous variables and as frequencies and percentages for categorical variables. Independent sample t tests, Pearson’s chi-square tests or Fisher’s exact test were used to analyze the differences in continuous variables and categorical variables between two groups. Multiple logistic regression analyses were used to estimate the ORs and 95% CIs for LGA in different BMI categories, and the results are presented as odds ratios (ORs) with 95% confidence intervals (CIs). To prove whether there were serial multiple mediation effects of FPG and mTG between prepregnancy BMI and LGA, we used R software to calculate these effects. The envisioned schema diagram is shown in Fig. 2 below. The ideograph of the total (Path c), direct (Path c’), and indirect (Path a1, a2, a3, b1, b2) effects of the association between the exposure (X) and the outcome (Y). To test whether indirect effects exist, we set the bootstrap confidence interval (CI) to 95%, and the number of bootstrap samples was 5,000. If zero was not included in the 95% CI interval, it indicated that the mediating effect was significant. The total effect of prepregnancy BMI categories on LGA was then calculated by summing the direct effect and the total indirect effect of BMI category. The percent mediation for each indirect effect was then calculated by dividing the indirect effect of each mediator by the total effect (direct and total indirect effects) of prepregnancy BMI. All statistical analyses were conducted using R version 4.1.2. All analysis p values < 0.05 were defined as significantly different.

## Results

### Maternal and infant characteristics

We finally included 19,104 women, 1228 of whom delivered LGA infants. Table 1 shows the maternal and infant characteristics of our study population. The prevalence of LGA in this research was 6.4%. The proportion of maternal age ≥ 35 years was 32.2% in mothers with LGA infants and 19.7% in the control group. As expected, the prepregnancy BMI of mothers delivering LGA infants was significantly higher than that of mothers in the control group (22.0 ± 2.8 vs. 20.8 ± 2.6, p < 0.001). In addition, there was a greater proportion of overweight/obese women with LGA (18.6% vs. 9.4%, 3.6% vs. 1.4%, p < 0.001). The incidence of cesarean section was more frequent in mothers with LGA infants than in mothers with non-LGA infants (62.2% vs. 35.1%, p < 0.001). Multiparous women had a higher proportion of LGA than primiparous pregnant women (60.3% vs. 39.7%, p < 0.001). Mothers with LGA infants had higher gestational weight gain than mothers in the control group (15.3 ± 4.3 vs. 13.7 ± 4.1, p < 0.001). There were higher birth weights among women with LGA (3976.8 ± 272.6 vs. 3258.0 ± 341.8, p < 0.001). There were no statistically significant differences between the LGA and non-LGA groups in maternal educational years, alcohol consumption, smoking, previous GDM history, nationality or family history of diabetes (p = 0.482; p = 0.616; p = 0.132; p = 0.202; p = 0.805; p = 0.476, respectively).

**Table 1:** Maternal and infant characteristic distributions among LGA and non-LGA infants

Meternal and Infant Characteristic	Total births n = 19,104	Non-LGA n = 17,876	LGA n = 1228	p
**BMI, mean(SD)**	20.8 (2.6)	20.8 (2.6)	22.0 (2.8)	< 0.001
**BMI group, n (%)**				
Underweight	3169 (16.6%)	3086 (17.3%)	83 (6.8%)	< 0.001
Normal	13,735 (71.9%)	12,863 (72.0%)	872 (71.0%)	
Overweight	1904 (10.0%)	1675 (9.4%)	229 (18.6%)	
Obese	296 (1.5%)	252 (1.4%)	44 (3.6%)	
**Maternal age, n (%)**				< 0.001
< 35	15,181 (79.5%)	14,348 (80.3%)	833 (67.8%)	
≥ 35	3923 (20.5%)	3528 (19.7%)	395 (32.2%)	
**Alcohol drinking, n (%)**				0.616
No	18,832(98.6%)	17,624 (98.6%)	1208 (98.4%)	
Yes	272 (1.4%)	252 (1.4%)	20 (1.6%)	
**Smoking, n (%)**				0.132
No	19,094 (99.9%)	17,868 (99.96%)	1226 (99.8%)	
Yes	10 (0.1%)	8 (0.04%)	2 (0.2%)	
**Parity, n (%)**				< 0.001
Primipara	10,945 (57.3%)	10,457 (58.5%)	488 (39.7%)	
Multipara	8159 (42.7%)	7491 (41.5%)	740 (60.3%)	
**Education, n (%)**				0.482
≤ 12 years	8194 (42.9%)	7655 (42.8%)	539 (43.9%)	
> 12 years	10,910 (57.1%)	10,221 (57.2%)	689 (56.1%)	
**Delivery mode, n (%)**				< 0.001
Vaginal delivery	12,061 (63.1%)	11,597 (64.9%)	464 (37.8%)	
Cesarean delivery	7043 (36.9%)	6279 (35.1%)	764 (62.2%)	
**Gestational weight gain, mean (SD)**	13.8 (4.1)	13.7 (4.1)	15.3 (4.3)	<0.001
**Family history of diabetes, n (%)**				0.476
No	18,226 (95.4%)	17,060 (95.4%)	1166 (95.0%)	
Yes	878 (4.6%)	816 (4.6%)	62 (5.0%)	
**Previous GDM history, n (%)**				0.202
No	19,091(99.9%)	17,865(99.9%)	1226(99.8%)	
Yes	13 (0.1%)	11 (0.1%)	2 (0.2%)	
**Nationality, n (%)**				0.805
Han	18,405 (96.3%)	17,224 (96.4%)	1181 (96.2%)	
Others	699 (3.7%)	652 (3.6%)	47 (3.8%)	
**Infant sex, n (%)**				< 0.001
Male	9934 (52.0%)	9072 (50.7%)	862 (70.2%)	
Female	9170 (48.0%)	8804 (49.3%)	366 (29.8%)	
**Neonatal BW, mean (SD)**	3304.2 (381.0)	3258.0 (341.8)	3976.8 (272.6)	< 0.001

### Maternal prepregnancy BMI and the risk of LGA

A logistic regression model was fitted to explore the relationship between maternal BMI category and the risk of LGA (Table 2). Adjustments were made for maternal age, education years, gestational weight gain, delivery mode, family history of diabetes, previous GDM history, nationality and parity. Women with underweight in reference to normal prepregnancy BMI had lower odds for LGA (adjusted odds ratios (AOR) = 0.47; 95% confidence intervals (CI) = 0.37–0.60). In contrast, women with overweight or obesity prepregnancy BMI had higher odds for LGA (AOR = 1.88; 95% CI = 1.60–2.21, AOR = 2.72; 95% CI = 1.93–3.84, respectively). After further adjustment for fasting plasma glucose level and maternal triglycerides from 24 to 28 weeks, the odds ratios for LGA according to maternal BMI were 0.51 (95% CI 0.41–0.65) for underweight mothers and 1.71 (95% CI 1.46–2.01) for overweight mothers. The offspring of mothers who were obese (AOR 2.33, 95% CI 1.64–3.29) had a significantly higher risk of LGA.

**Table 2:** Logistic regression of the correlation between maternal prepregnancy BMI categories and LGA

BMI Category	Model 1		Model 2	
	Adjusted odds ratio (95%CI)	P	Adjusted odds ratio (95%CI)	P
Underweight	0.47 (0.37, 0.60)	< 0.001	0.51 (0.41, 0.65)	< 0.001
Normal	Ref.	-	Ref.	-
Overweight	1.88 (1.60, 2.21)	< 0.001	1.71 (1.46, 2.01)	< 0.001
Obesity	2.72 (1.93, 3.84)	< 0.001	2.33 (1.64, 3.29)	< 0.001

Model 1, adjusted for maternal age, education years, gestational weight gain, delivery mode, family history of diabetes, previous GDM history, nationality and parity; Model 2, further adjusted for fasting plasma glucose level and maternal triglycerides based on Model 1

### Testing for the significance of designed model

The analysis results are presented in Table 3. The association between overweight/obesity and FPG was significant, as the Path a1 effect was 0.087 (95% CI, 0.072–0.102) and 0.153 (95% CI, 0.116–0.190). The association between overweight/obesity and mTG was also significant, as the Path a2 effect was 0.243 (95% CI, 0.204–0.283) and 0.349 (95% CI, 0.256–0.442). In both overweight and obese individuals, FPG was related to mTG due to Path a3 effects of 0.232 (95% CI, 0.192–0.272) and 0.217 (95% CI, 0.175–0.259), respectively. Path b1, with effects of 0.042 (95% CI, 0.029–0.054) and 0.039 (95% CI, 0.027–0.052), confirmed that FPG was significantly associated with LGA in both overweight and obese women. In addition, mTG also had a relationship with the occurrence of LGA, with path b2 effects of 0.014 (95% CI, 0.009–0.019) and 0.016 (95% CI, 0.011–0.021) among overweight and obese women, respectively.

**Table 3:** Testing for the designed model of the relationship between prepregnancy overweight/obesity and LGA

Pathway	Overweight					Obesity			
	Effect	SE	LLCI	ULCI		Effect	SE	LLCI	ULCI
Path a1	0.087	0.008	0.072	0.102		0.153	0.019	0.116	0.190
Path a2	0.243	0.020	0.204	0.283		0.349	0.048	0.256	0.442
Path a3	0.232	0.021	0.192	0.272		0.217	0.021	0.175	0.259
Path b1	0.042	0.006	0.029	0.054		0.039	0.007	0.027	0.052
Path b2	0.014	0.002	0.009	0.019		0.016	0.003	0.011	0.021

Path a1-3 and Path b1-2 are all adjusted for maternal age, education years, gestational weight gain, delivery mode, family history of diabetes, previous GDM history, nationality and parity

### Serial multiple mediation analysis of prepregnancy BMI and LGA

The serial multiple mediation analysis results of prepregnancy BMI and LGA on FPG and mTG are shown in Table 4. The estimates of the total, direct and indirect effects in the association with maternal overweight/obesity on fetal LGA with FPG and mTG at 24–28 weeks as mediators were significant. All three indirect and direct paths were also significant in overweight and obese women. Specifically, in prepregnancy overweight women, the total effect (Path c) of overweight on LGA was 0.051 (95% CI, 0.035–0.065; p < 0.001), including a direct effect pathway (Path c’) of 0.043 (95% CI, 0.028–0.058; p < 0.001) and three indirect effect pathways. The first indirect pathway mediated the effect of prepregnancy overweight on LGA mediated by FPG, with an effect value of 0.004 (95% CI, 0.002–0.005; p < 0.001). The second indirect pathway was significantly mediated by mTG, with an effect value of 0.003 (95% CI, 0.002–0.005; p < 0.001). The third indirect pathway was that the effect of prepregnancy overweight on LGA was significantly mediated by both FPG and mTG, with an effect value of 0.000 (95% CI, 0.000–0.000; p < 0.001). The mediated proportions were 7.8%, 5.9%, and 0% through the three indirect pathways. Additionally, prepregnancy obesity not only had a direct positive effect (Path c’) on LGA with an effect value of 0.076 (95% CI, 0.037–0.118; p < 0.001) but also had an indirect effect on LGA through three paths: the independent mediating role of FPG (effect = 0.006; 95% CI: 0.004–0.009); the independent mediating role of mTG (effect = 0.006; 95% CI: 0.003–0.008); and the chain mediating role of FPG and mTG (effect = 0.001; 95% CI: 0.000-0.001). The total effect (Path c) of prepregnancy obesity on LGA was 0.089 (95% CI, 0.050–0.130; p < 0.001). The estimated proportions were 6.7%, 6.7%, and 1.1%, respectively.

**Table 4:** Mediating effects of FPG/mTG on the association between prepregnancy overweight/obesity and fetal LGA

BMI Category	Total Effect (c) (95% CI)	Direct Effect (c’) (95% CI)	Indirect 1			Indirect 2			Indirect 3	
			Indirect Effect (95% CI)	Mediated Proportion		Indirect Effect (95% CI)	Mediated Proportion		Indirect Effect (95% CI)	Mediated Proportion
Overweight	0.051*** (0.035, 0.065)	0.043*** (0.028, 0.058)	0.004*** (0.002, 0.005)	7.8%		0.003*** (0.002, 0.005)	5.9%		0.000*** (0.000, 0.000)	0%
Obesity	0.089*** (0.050, 0.130)	0.076*** (0.037, 0.118)	0.006*** (0.004, 0.009)	6.7%		0.006*** (0.003, 0.008)	6.7%		0.001*** (0.000, 0.001)	1.1%

Indirect 1, Overweight/Obesity → FPG → LGA; Indirect 2, Overweight/Obesity → mTG → LGA; Indirect 3, Overweight/Obesity → FPG→ mTG → LGA; * p < 0.05, ** p < 0.01, *** p < 0.001

## Discussion

In our population-based study, we explored the relationship of prepregnancy BMI with LGA in Chinese reproductive-age women. Previous studies have shown that prepregnancy maternal underweight increased the risk of low birth weight babies, while overweight or obesity increased the risk of large size babies [26]. Similarly, according to a cohort study from Poland, compared with normal BMI, maternal obesity was associated with a higher risk of macrosomia after adjusting for potential confounders, and the result was still maintained in the subgroup of ‘healthy’ women, namely, women who did not develop either diabetes or hypertension in the current pregnancy [27]. This is consistent with the findings of our study population, as our study population also excluded pregnant women with diabetes and hypertension, either diagnosed before or during pregnancy.

Our study evaluated the mediating effects of maternal fasting glucose and triglycerides simultaneously to discern which markers had the greatest individual mediating effects in nondiabetic pregnant women. Interestingly, fasting glucose levels and triglycerides were found to mediate the effect of maternal obesity on the occurrence of LGA in the same proportion, and the mediated proportion was 6.7%, indicating that both are important for the effect of maternal obesity on LGA. With this analysis, we provide data on how maternal circulating energy substances, including fasting glucose levels and triglycerides, mediate the association between maternal overweight/obesity and LGA in nondiabetic pregnancies. Based on these studies, it is suggested that circulating lipid levels, such as triglycerides, are as important as glucose levels in influencing birth weight in women with overweight/obesity.

Fetal growth depends on maternal factors (including maternal health status, nutritional status, smoking, drug use, etc.), fetal factors (genetic composition) and placental function [28]. The delivery of excess maternal nutrients to the fetus is known to increase the risk of macrosomia, even among infants of women without gestational diabetes mellitus [29]. LGA infants may have increased intrauterine exposure to excess nutrients, especially glucose, which can lead to hyperinsulinemia, increased use of oxygen and glucose, and oxidative stress, resulting in increased lipolysis and reduced insulin sensitivity at birth [30, 31]. Fetal metabolic abnormalities may lead to complications such as hypoglycemia, polycythemia and asphyxia [31]. LGA offspring of diabetic mothers were at significant risk of developing metabolic syndrome in childhood with a predisposition to develop cardiovascular disease and diabetes in adulthood [32].

Pedersen’s hypothesis is that elevated maternal blood sugar levels can cross the placenta and stimulate the secretion of insulin by fetal islet cells, leading to hyperinsulinemia. Increasing glucose utilization, promoting protein and fat synthesis and inhibiting lipolysis lead to fetal weight gain [33]. In addition, pregnant women are prone to abnormal lipid metabolism during pregnancy. Maternal lipoproteins do not cross the placenta but are hydrolyzed by placental lipoprotein lipase. Derived fatty acids in pregnant women enter the fetal blood circulation through the placenta from the mother and then take up synthesized triglycerides through the fetal liver, which can promote fetal fat accumulation [34, 35]. maternal lipid concentrations may exert in utero influences on infant body composition [36].

Maternal prepregnancy overweight/obesity appears to be the most influential upstream metabolic risk factor affecting maternal and neonatal metabolic health [37]. In addition, maternal glucose levels have been extensively studied and are believed to have a significant effect on offspring birth weight even in the absence of maternal diabetes [38]. Using a secondary analysis of a subgroup of women in the HAPO study, the study found that a higher maternal BMI category was associated with higher odds of neonatal birth weight and obesity and that fasting glucose metabolism was the strongest mediator of the association between maternal BMI and neonatal size and weight during OGTT at 24–32 weeks of gestation in normal-glycemic pregnancies [39]. The effect of maternal overweight or obesity on larger offspring size has been attributed mainly to higher pregnancy glucose levels [38].

In addition, the lipid profile suffers adaptive changes during pregnancy due to estrogen stimulation and insulin resistance. Studies have shown that prepregnancy BMI and fasting serum TG were independently associated with birth weight in normal glucose tolerance mothers [40]. Lu et al. reported that mTG mediates the association of prepregnancy BMI with the risk of fetal macrosomia, which highlights the importance of the role that maternal circulating mTG during pregnancy plays in the development of fetal macrosomia [41]. Similarly, Song et al. found that nondiabetic women who were overweight/obese before pregnancy were at increased risk of developing a giant fetus, and this positive association was mediated in part by high maternal TG levels [42].

At present, ultrasonic measurement and clinical evaluation based on maternal uterine height are used to predict fetal weight. Some studies have suggested using two-dimensional ultrasound during the second trimester to measure fetal epicardial adipose tissue and the visceral fat structure between the right ventricular pericardium and the heart muscle to predict the development of macrosomia [43]. Fetal epicardial fat thickness is a focus of concern in diabetic pregnancy and is also considered an indicator of fetal growth [44].

Our study found that prepregnancy overweight/obesity increased the risk of birth LGA and was partially mediated by FPG and TG levels from 24 to 28 weeks of gestation. In addition, studies have also found that 50 g GCT can be used to identify women at risk of delivering overweight infants or small for gestational age neonates [45, 46]. Excess birth weight has been found to be associated with increased 50 g GCT values in pregnant women delivering large for gestational age babies [46]. In Finland and Poland, 50 g GCT is preferred for universal screening of GDM [47]. It is economical, practical and tolerable to patients. These screening tests may provide useful information about the growth potential of the fetus, helping to identify those at high risk for LGA birth. We also need to explore more ways to predict the occurrence of LGA and intervene early.

This finding implies that pregnant women who are overweight or obese before pregnancy may benefit from the management of circulating mTG to prevent fetal overgrowth. Clinicians help guide pregnant women in achieving reasonable dietary nutrition during pregnancy and reduce patients’ sugar intake on the premise of ensuring maternal and infant nutritional needs [48]. In addition, women without contraindications should be encouraged to engage in aerobic exercise and strength training during pregnancy to reduce the risk of macrosomia [1]. Clinicians also need to closely monitor the glycemic and lipid levels of pregnant women, especially TG, FBG and other indicators, to effectively provide guidance and intervention in advance.

This study involved a large sample size of people, and maternal BMI was calculated on the basis of weight and height measured in early pregnancy, which reduces the risks of recall and selection bias. This serial multiple mediation simultaneously assessed the mediating effect of FPG and mTG in the associations between maternal overweight/obesity and LGA, which helps to discern markers that exhibit a greater individual mediating effect. However, several limitations of the study should be acknowledged. We analyzed fasting blood glucose and triglycerides only in the second trimester of pregnancy and did not perform a longitudinal assessment of dynamic changes throughout pregnancy. We failed to include sufficient information on dietary nutrition intake. Thus, additional studies are needed to clarify the relationship between maternal dietary structure and pregnancy outcome. Due to the floating population in Shenzhen and incomplete data collection on LGA history in pregnant women, we have not included information about LGA history. In the future, we need to further explore other ways through which maternal BMI leads to the occurrence of macrosomia to find controllable factors to reduce the occurrence of macrosomia.

## Conclusions

This study found that in nondiabetic women, maternal overweight/obesity was positively associated with the occurrence of LGA, and this positive association was partly mediated by FPG and mTG, emphasizing the important role of maternal overweight/obesity and the potential role of maternal circulating fuels in fetal growth. Both FPG and mTG were strong mediators of these associations, suggesting that mTG is as important as FPG and deserves the attention of clinicians. Future close monitoring of triglycerides during pregnancy may be required in the care of women who are overweight or obese during pregnancy even without a diagnosis of diabetes.

## Data Availability

The data presented in this study are available on request from the corresponding author.

## Abbreviations

FPG: Fasting plasma glucose
mTG: Maternal triglyceride
LGA: Large for gestational age
GDM: Gestational diabetes mellitus
NICHD: National Institute of Child Health and Human Development
IADPSG: International Association of Diabetes and Pregnancy Study Groups
OGTT: Oral Glucose Tolerance Test
BMI: Body mass index
BW: Birth weight
SGA: Small for gestational age
SE: Standard error
LLCI: Lower limit confidence interval
ULCI: Upper limit confidence interval

## References

[1] Macrosomia (2020). ACOG Practice Bulletin, Number 216. Obstet Gynecol.

[2] Gyselaers W, Martens G (2012). Increasing prevalence of macrosomia in Flanders, Belgium: an indicator of population health and a burden for the future. Facts Views Vis Obgyn.

[3] Araujo Júnior E, Peixoto AB, Zamarian AC, Elito Júnior J, Tonni G (2017). Macrosomia. Best Pract Res Clin Obstet Gynaecol.

[4] Nguyen MT, Ouzounian JG (2021). Evaluation and management of fetal macrosomia. Obstet Gynecol Clin North Am.

[5] Sparano S, Ahrens W, De Henauw S, Marild S, Molnar D, Moreno LA, Suling M, Tornaritis M, Veidebaum T, Siani A (2013). Being macrosomic at birth is an independent predictor of overweight in children: results from the IDEFICS study. Matern Child Health J.

[6] Hermann GM, Dallas LM, Haskell SE, Roghair RD (2010). Neonatal macrosomia is an independent risk factor for adult metabolic syndrome. Neonatology.

[7] Gulecoglu Onem MG, Coker C, Baysal K, Altunyurt S, Keskinoglu P (2021). The effects of pre-pregnancy obesity and gestational weight gain on maternal lipid profiles, fatty acids and insulin resistance. J Perinat Med.

[8] Ramsay JE, Ferrell WR, Crawford L, Wallace AM, Greer IA, Sattar N (2002). Maternal obesity is associated with dysregulation of metabolic, vascular, and inflammatory pathways. J Clin Endocrinol Metab.

[9] Owens LA, O’Sullivan EP, Kirwan B, Avalos G, Gaffney G, Dunne F (2010). ATLANTIC DIP: the impact of obesity on pregnancy outcome in glucose-tolerant women. Diabetes Care.

[10] Yang Z, Phung H, Freebairn L, Sexton R, Raulli A, Kelly P (2019). Contribution of maternal overweight and obesity to the occurrence of adverse pregnancy outcomes. Aust N Z J Obstet Gynaecol.

[11] Kc K, Shakya S, Zhang H (2015). Gestational diabetes mellitus and macrosomia: a literature review. Ann Nutr Metab.

[12] Metzger BE, Lowe LP, Dyer AR, Trimble ER, Chaovarindr U, Coustan DR, Hadden DR, McCance DR, Hod M, McIntyre HD (2008). Hyperglycemia and adverse pregnancy outcomes. N Engl J Med.

[13] Catalano PM, McIntyre HD, Cruickshank JK, McCance DR, Dyer AR, Metzger BE, Lowe LP, Trimble ER, Coustan DR, Hadden DR (2012). The hyperglycemia and adverse pregnancy outcome study: associations of GDM and obesity with pregnancy outcomes. Diabetes Care.

[14] Tennant P, Doxford-Hook E, Flynn L, Kershaw K, Goddard J, Stacey T (2022). Fasting plasma glucose, diagnosis of gestational diabetes and the risk of large for gestational age: a regression discontinuity analysis of routine data. BJOG.

[15] Bevier WC, Fischer R, Jovanovic L (1999). Treatment of women with an abnormal glucose challenge test (but a normal oral glucose tolerance test) decreases the prevalence of macrosomia. Am J Perinatol.

[16] Olmos PR, Borzone GR, Olmos RI, Valencia CN, Bravo FA, Hodgson MI, Belmar CG, Poblete JA, Escalona MO, Gómez B (2012). Gestational diabetes and pre-pregnancy overweight: possible factors involved in newborn macrosomia. J Obstet Gynaecol Res.

[17] Schaefer-Graf UM, Graf K, Kulbacka I, Kjos SL, Dudenhausen J, Vetter K, Herrera E (2008). Maternal lipids as strong determinants of fetal environment and growth in pregnancies with gestational diabetes mellitus. Diabetes Care.

[18] Herrera E, Desoye G (2016). Maternal and fetal lipid metabolism under normal and gestational diabetic conditions. Horm Mol Biol Clin Investig.

[19] Olmos PR, Rigotti A, Busso D, Berkowitz L, Santos JL, Borzone GR, Poblete JA, Vera C, Belmar C, Goldenberg D (2014). Maternal hypertriglyceridemia: a link between maternal overweight-obesity and macrosomia in gestational diabetes. Obes (Silver Spring).

[20] Xi F, Chen H, Chen Q, Chen D, Chen Y, Sagnelli M, Chen G, Zhao B, Luo Q (2021). Second-trimester and third-trimester maternal lipid profiles significantly correlated to LGA and macrosomia. Arch Gynecol Obstet.

[21] Vrijkotte TG, Krukziener N, Hutten BA, Vollebregt KC, van Eijsden M, Twickler MB (2012). Maternal lipid profile during early pregnancy and pregnancy complications and outcomes: the ABCD study. J Clin Endocrinol Metab.

[22] Furse S, White SL, Meek CL, Jenkins B, Petry CJ, Vieira MC, Ozanne SE, Dunger DB, Poston L, Koulman A (2019). Altered triglyceride and phospholipid metabolism predates the diagnosis of gestational diabetes in obese pregnancy. Mol Omics.

[23] Metzger BE, Gabbe SG, Persson B, Buchanan TA, Catalano PA, Damm P, Dyer AR, Leiva A, Hod M, Kitzmiler JL (2010). International association of diabetes and pregnancy study groups recommendations on the diagnosis and classification of hyperglycemia in pregnancy. Diabetes Care.

[24] He W, Li Q, Yang M, Jiao J, Ma X, Zhou Y, Song A, Heymsfield SB, Zhang S, Zhu S (2015). Lower BMI cutoffs to define overweight and obesity in China. Obes (Silver Spring).

[25] Buck Louis GM, Grewal J, Albert PS, Sciscione A, Wing DA, Grobman WA, Newman RB, Wapner R, D’Alton ME, Skupski D et al. Racial/ethnic standards for fetal growth: the NICHD Fetal Growth Studies. *Am J Obstet Gynecol* 2015, 213(4):449.e441-449.e441. 10.1016/j.ajog.2015.08.032 PMC4584427 26410205

[26] Gondwe A, Ashorn P, Ashorn U, Dewey KG, Maleta K, Nkhoma M, Mbotwa J, Jorgensen JM (2018). Pre-pregnancy body mass index (BMI) and maternal gestational weight gain are positively associated with birth outcomes in rural Malawi. PLoS ONE.

[27] Lewandowska M. Maternal obesity and risk of low Birth Weight, fetal growth restriction, and Macrosomia: multiple analyses. Nutrients 2021, 13(4). 10.3390/nu13041213 PMC8067544 33916963

[28] Maulik D (2006). Fetal growth restriction: the etiology. Clin Obstet Gynecol.

[29] Retnakaran R, Ye C, Hanley AJ, Connelly PW, Sermer M, Zinman B, Hamilton JK (2012). Effect of maternal weight, adipokines, glucose intolerance and lipids on infant birth weight among women without gestational diabetes mellitus. CMAJ.

[30] Akinbi HT, Gerdes JS (1995). Macrosomic infants of nondiabetic mothers and elevated C-peptide levels in cord blood. J Pediatr.

[31] Ahlsson FS, Diderholm B, Ewald U, Gustafsson J (2007). Lipolysis and insulin sensitivity at birth in infants who are large for gestational age. Pediatrics.

[32] Scifres CM (2021). Short- and Long-Term Outcomes Associated with large for gestational age Birth Weight. Obstet Gynecol Clin North Am.

[33] Pedersen J. Diabetes mellitus and pregnancy: present status of the hyperglycaemia–hyperinsulinism theory and the weight of the newborn baby. Postgrad Med J 1971:Suppl:66–7. 5547509

[34] Hashemipour S, Haji Seidjavadi E, Maleki F, Esmailzadehha N, Movahed F, Yazdi Z (2018). Level of maternal triglycerides is a predictor of fetal macrosomia in non-obese pregnant women with gestational diabetes mellitus. Pediatr Neonatol.

[35] Herrera E, Ortega-Senovilla H (2018). Implications of lipids in neonatal body weight and Fat Mass in Gestational Diabetic Mothers and non-diabetic controls. Curr Diab Rep.

[36] Geraghty AA, Alberdi G, O’Sullivan EJ, O’Brien EC, Crosbie B, Twomey PJ, McAuliffe FM (2016). Maternal blood lipid Profile during pregnancy and Associations with Child Adiposity: findings from the ROLO Study. PLoS ONE.

[37] Wang J, Kuang Y, Shen S, Price MJ, Lu J, Sattar N, He J, Pittavino M, Xia H, Thomas GN (2022). Association of maternal lipid levels with birth weight and cord blood insulin: a bayesian network analysis. BMJ Open.

[38] Ong KK, Diderholm B, Salzano G, Wingate D, Hughes IA, MacDougall J, Acerini CL, Dunger DB (2008). Pregnancy insulin, glucose, and BMI contribute to birth outcomes in nondiabetic mothers. Diabetes Care.

[39] Andrews C, Monthé-Drèze C, Sacks DA, Ma RCW, Tam WH, McIntyre HD, Lowe J, Catalano P, Sen S (2021). Role of maternal glucose metabolism in the association between maternal BMI and neonatal size and adiposity. Int J Obes (Lond).

[40] Di Cianni G, Miccoli R, Volpe L, Lencioni C, Ghio A, Giovannitti MG, Cuccuru I, Pellegrini G, Chatzianagnostou K, Boldrini A (2005). Maternal triglyceride levels and newborn weight in pregnant women with normal glucose tolerance. Diabet Med.

[41] Lu S, Fu Y, Wu YY, Mao AF, Xu MY, Zheng G, Cai FC, Wang XH, Shi MQ, Hu WS (2020). Mediating Effects of maternal blood triglycerides on the relationship between Prepregnancy Body Mass Index and fetal macrosomia. J Pediatr.

[42] Song X, Chen L, Zhang S, Liu Y, Wei J, Sun M, Shu J, Wang T, Qin J. High maternal triglyceride levels mediate the Association between Pre-Pregnancy Overweight/Obesity and macrosomia among Singleton Term non-diabetic pregnancies: a prospective cohort study in Central China. Nutrients 2022, 14(10). 10.3390/nu14102075 PMC9145691 35631216

[43] Aydin E, Tanacan A, Bulut AN (2021). A cut-off value of epicardial fat thickness for the prediction of large for gestational age foetuses. J Obstet Gynaecol.

[44] Jackson D, Deschamps D, Myers D, Fields D, Knudtson E, Gunatilake R (2016). Fetal epicardial fat thickness in diabetic and non-diabetic pregnancies: a retrospective cross-sectional study. Obes (Silver Spring).

[45] Tanacan A, Eyupoglu M, Fadiloglu E, Zengin HY, Karaagaoglu E, Beksac MS (2020). Use of the 50-g glucose challenge test to predict small-for-gestational-age neonates. J Diabetes.

[46] Beksac MS, Tanacan A, Hakli DA, Ozyuncu O (2018). Use of the 50-g glucose challenge test to predict excess delivery weight. Int J Gynaecol Obstet.

[47] Petrović O, Belci D (2017). A critical appraisal and potentially new conceptual approach to screening and diagnosis of gestational diabetes. J Obstet Gynaecol.

[48] Guillemette L, Durksen A, Rabbani R, Zarychanski R, Abou-Setta AM, Duhamel TA, McGavock JM, Wicklow B (2017). Intensive gestational glycemic management and childhood obesity: a systematic review and meta-analysis. Int J Obes (Lond).

